# Ecological Divergence Within the Enterobacterial Genus *Sodalis*: From Insect Symbionts to Inhabitants of Decomposing Deadwood

**DOI:** 10.3389/fmicb.2021.668644

**Published:** 2021-06-11

**Authors:** Vojtěch Tláskal, Victor Satler Pylro, Lucia Žifčáková, Petr Baldrian

**Affiliations:** ^1^Laboratory of Environmental Microbiology, Institute of Microbiology of the Czech Academy of Sciences, Praha, Czechia; ^2^Microbial Ecology and Bioinformatics Laboratory, Department of Biology, Federal University of Lavras (UFLA), Lavras, Brazil

**Keywords:** insect symbionts, *Sodalis*, deadwood, nitrogen fixation, free-living, non-symbiotic

## Abstract

The bacterial genus *Sodalis* is represented by insect endosymbionts as well as free-living species. While the former have been studied frequently, the distribution of the latter is not yet clear. Here, we present a description of a free-living strain, *Sodalis ligni* sp. nov., originating from decomposing deadwood. The favored occurrence of *S. ligni* in deadwood is confirmed by both 16S rRNA gene distribution and metagenome data. Pangenome analysis of available *Sodalis* genomes shows at least three groups within the *Sodalis* genus: deadwood-associated strains, tsetse fly endosymbionts and endosymbionts of other insects. This differentiation is consistent in terms of the gene frequency level, genome similarity and carbohydrate-active enzyme composition of the genomes. Deadwood-associated strains contain genes for active decomposition of biopolymers of plant and fungal origin and can utilize more diverse carbon sources than their symbiotic relatives. Deadwood-associated strains, but not other *Sodalis* strains, have the genetic potential to fix N_2_, and the corresponding genes are expressed in deadwood. Nitrogenase genes are located within the genomes of *Sodalis*, including *S. ligni*, at multiple loci represented by more gene variants. We show decomposing wood to be a previously undescribed habitat of the genus *Sodalis* that appears to show striking ecological divergence.

## Introduction

Bacterial insect endosymbionts that occupy tissues of their hosts are widespread in multiple insect taxa (reviewed in Perlmutter and Bordenstein, 2020). The nature of such symbiosis may take the form of parasitism, mutualism or commensalism depending on the ecology of the bacterial symbiont. Parasitic bacteria may manipulate reproduction of their hosts, while mutualistic bacteria may provide essential vitamins or participate in nitrogen (N) cycling (Akman et al., 2002; Behar et al., 2005; Sabree et al., 2009). Commensal bacteria can use insects at some part of their life cycle as vectors for transmission to their target host organism, e.g., plants for which they are parasitic (Nadarasah and Stavrinides, 2011). The life strategy of the symbiont depends on the evolutionary history with the host and is driven by the length of its coexistence with the host (Toh et al., 2006). Different bacterial symbionts with distinct evolutionary histories have been described, e.g., in tsetse flies (*Glossina* sp.). *Wigglesworthia glossinidia* (*Enterobacterales*) provides vitamins to the tsetse fly and to other symbionts (Hall et al., 2019) and represents an obligate mutualist with a long coevolutionary history (Chen et al., 1999). Another tsetse fly enterobacterium, *Sodalis glossinidius*, shows a more recent association with its host as suggested by its relatively large genome that contains a high number of pseudogenes, which remain actively transcribed in a cell-free culture (Toh et al., 2006; Goodhead et al., 2020; Hall et al., 2020). Further hinting at the recent establishment of the *Sodalis*-tsetse symbiosis was the successful establishment and vertical transmission of the non-native *Sodalis* population in tsetse flies cleared from their native symbionts. It indicates lack of host-symbiont extensive coevolution (Weiss et al., 2006).

*Sodalis* endosymbionts are present in other insects with nutritionally restricted diets, such as bloodsucking Diptera (Nováková and Hypša, 2007; Chrudimský et al., 2012), Phthiraptera (Fukatsu et al., 2007; Boyd et al., 2016), sapsucking Hemiptera (Kaiwa et al., 2010) and Coleoptera (Heddi et al., 1999). Multiple *Sodalis* species have thus been identified within their hosts and shown to supply essential vitamins to the host (Heddi et al., 1999; Boyd et al., 2016). In contrast, only one free-living member of this genus has been described despite the apparent recent establishment of the host-dependent life strategy of the endosymbionts. *Sodalis* sp. strain HS, later described as *S. praecaptivus* was isolated from a hand wound of a human impaled with a tree branch (Clayton et al., 2012; Chari et al., 2015). Incidental isolation of *Sodalis-*related strain from such source was unexpected and as a consequence *Sodalis* became a textbook example of microbial specialization processes (Yong, 2016).

The genome-wide sequence comparison of *Sodalis* species shows *S. glossinidius* and the cereal weevil symbiont *S. pierantonius* strain SOPE to be closely related to *S. praecaptivus.* In particular, the *S. pierantonius* strain SOPE shares high genome similarity with free-living *S. praecaptivus*, and its symbiosis with the weevil shows evidence of recent origin (Clayton et al., 2012). Despite the presence of numerous pseudogenes, genes of both symbionts represent a subset of the genes in the *S. praecaptivus* genome, showing that the *S. praecaptivus*-like ancestor might be the ancestor for symbionts as well (Clayton et al., 2012). The genome characteristics of *S. praecaptivus* correspond to host-independent life and stabilizing gene selection pressure; in comparison with symbiotic relatives, it has a larger genome, fewer pseudogenes and fewer IS elements (Oakeson et al., 2014). Consequently, due to its wide spectrum of genes and high coding density, *S. praecaptivus* is able to utilize several carbon (C) sources, including chitin and plant sugars (cellobiose, xylose, rhamnose) and N sources, such as ammonia and nitrate (Chari et al., 2015). While *S. praecaptivus* is host-independent, it is able to colonize weevils and tsetse flies through quorum sensing suppression of its virulence, which suggests a mechanism of insect symbiosis development in the *Sodalis* group (Enomoto et al., 2017; Munoz et al., 2020). Despite the well-characterized phylogeny and genome properties of the *Sodalis* group, a systematic approach to assess the environmental distribution of free-living *Sodalis* strains has not been performed. It remains to be determined whether there are different levels of relatedness of non-symbiotic strains with symbionts in distinct insect taxa hosts.

Here, we present a *Sodalis* pangenome analysis that allowed us to characterize different guilds within this genus. Guilds differ in their gene composition as well as in their life strategy. Our results show that the *Sodalis* genus is functionally versatile with multiple ecological roles. We report novel free-living *Sodalis* species-level taxon associated in high abundances with decomposing wood, for which we propose the name *Sodalis ligni* sp. nov. This taxon is represented by the strain dw23^*T*^ and can utilize C-rich labile wood-derived compounds and fix atmospheric nitrogen. *Sodalis ligni* is a globally distributed species that is directly related to symbiotic *Sodalis* members with characterized genomes while maintaining a non-symbiotic lifestyle and preferring deadwood habitats.

## Materials and Methods

### *Sodalis* Isolation

Wood samples for bacterial isolation were obtained in the Žofínský Prales National Nature Reserve, an unmanaged forest in the south of the Czech Republic (48°39′57″N, 14°42′24″E). The core zone of the forest reserve (42 ha) has never been managed, and human intervention stopped in 1838, when it was declared a reserve. This reserve thus represents a rare fragment of European temperate virgin forest with deadwood left to spontaneous decomposition. The reserve is situated at 730–830 m a.s.l., the bedrock is almost homogeneous and consists of fine to medium-grainy porphyritic and biotite granite. The annual average rainfall is 866 mm, and the annual average temperature is 6.2°C (Anderson-Teixeira et al., 2015). At present, the reserve is covered by a mixed forest in which *Fagus sylvatica* predominates, followed by *Picea abies* and *Abies alba*. The mean living tree volume is 690 m^3^ h^–1^, and the mean volume of coarse woody debris (logs, represented by tree trunks and their fragments) is 208 m^3^ h^–1^ (Král et al., 2010; Šamonil et al., 2013). Logs are repeatedly surveyed, and the approximate age of each log, the cause of death (e.g., stem breakage, windthrow, etc.) and the status before downing (fresh, decomposed) is known. The area has been subjected to the characterization of bacterial and fungal succession on deadwood (Baldrian et al., 2016; Tláskal et al., 2017; Odriozola et al., 2021), and the functional roles of the members of the deadwood microbiome were determined (Tláskal et al., 2021).

Wood samples were obtained as described previously in Tláskal et al. (2017). Briefly, in October 2013, four samples from selected logs were obtained by drilling with an electrical drill vertically along the whole decomposing stem. Part of the material was used for bacterial community characterization (Tláskal et al., 2017) and part was treated as follows: wood chips from each stem were pooled together and transported to the laboratory, where they were kept at 4°C until the next day. Wood material was shaken with 15 mL of Ringer solution for 2 h and diluted 10^4^× to 10^6^×. Dilutions were plated on nutrient-limited NB medium (0.26 g L^–1^ Nutrient Broth, 15 g L^–1^ agar, pH 5). The growth of bacterial colonies was recorded on Petri dishes using a marker for 8 weeks. Colony PCR was used to infer the taxonomy of the slow-growing strains that appeared in the later phase of cultivation. The PCR premix and cycling conditions were as follows: 2.5 μL 10 × buffer for DyNAzyme DNA Polymerase; 0.75 μL DyNAzyme II DNA polymerase (2 μL^–1^); 0.75 μL of BSA (20 mg mL^–1^); 0.5 μL of PCR Nucleotide Mix (10 mM); 1 μL of each primer eub530f (10 μM) and eub1100br (10 μM) (Lane, 1991) and sterile ddH_2_O up to 25 μL; amplification started at 94°C for 5 min, followed by 35 cycles of 94°C for 1 min, 62°C for 1 min, 72°C for 1 min and finished with a final setting of 72°C for 10 min. Sanger sequencing was performed using the reverse primer. The obtained sequences were compared by BLASTn with bacterial 16S rRNA gene-based community data from the same habitat (Tláskal et al., 2017). Bacterial strains with high similarity and coverage to the most abundant bacteria recovered by environmental DNA sequencing were selected for further cultivation and genome sequencing (Tláskal and Baldrian, 2021).

By this approach, we were able to select two bacterial strains (labeled *Sodalis* sp. strain dw23^*T*^ and strain dw96) with high similarity to the abundant cluster CL27 in community data (Tláskal et al., 2017), which is 100% similar to the 16S rRNA gene sequence with NCBI accession AJ011333.1, which is mislabeled as *Yersinia* sp. (Elo et al., 2000). The nearest relative with a complete genome sequence available was the *Sodalis praecaptivus* strain HS (CP006569.1, similarity 97.2%, Chari et al., 2015). Strains dw23^*T*^ and dw96 originated from two distinct *Fagus sylvatica* stems 9 m and 12 m long, respectively, that had been decomposing for less than 5 years.

### DNA Extraction, Sequencing, and Genome Assembly

The two *Sodalis* strains were cultivated in 50 mL of GY-VL55 liquid medium (Lladó et al., 2019) with shaking for 2 weeks at 23°C. After cultivation, the cells were pelleted and DNA was extracted using the ArchivePure DNA Yeast & Gram- + Kit (5 Prime, Germany) according to the manufacturer’s instructions. The DNA was quantified by a Qubit 2.0 Fluorometer (Life Technologies, United States), and sheared by Bioruptor Pico (Diagenode, Belgium) to an average length of 550 bp. Sequencing adapters were ligated by the TruSeq DNA PCR-Free Library Prep Kit (Illumina Inc., United States). The ligated library was sequenced on the Illumina MiSeq platform with 2 × 250 (strain dw23^*T*^) and 2 × 300 (strain dw96) paired-end runs.

*Sodalis ligni* dw23^*T*^ was selected for further Nanopore MinION sequencing. DNA was extracted by mechanical cell lysis using vortex, followed by DNA binding and purification on AMPure XP magnetic beads (Beckman Coulter, United States) with 70% ethanol. The SQK-LSK108 ligation kit was used to prepare a long-read sequencing library according to the manufacturer’s instructions. The library was loaded onto a Nanopore flow-cell version FLO-MIN106 for a 48 h sequencing run. The obtained FAST5 reads were basecalled into FASTQ with local Albacore 2.1.7 (available via ONT community site^1^) with a minimal quality threshold of 7. Passed reads were scanned for remaining adapters, which were trimmed with Porechop 0.2.3^2^.

The *Sodalis* sp. strain dw96 genome assembly used short reads only, and *Sodalis ligni* dw23^*T*^ used a hybrid assembly of short and long reads. Assembly was performed with Unicycler 0.4.4 (Wick et al., 2017) in normal mode wrapping the following programs: SPAdes 3.11.1 (Bankevich et al., 2012), BLAST 2.2.28+ (Altschul et al., 1997), bowtie 2.2.4 (Langmead et al., 2009), samtools 1.6 (Li et al., 2009) and pilon 1.22 (Walker et al., 2014). Prokka 1.13 (Seemann, 2014) with RNAmmer (Lagesen et al., 2007) was used for gene calling, annotation and rRNA genes identification. Genome completeness and contamination were estimated using CheckM v1.1.3 (Parks et al., 2015). The genomes were compared with NCBI Prok database of complete prokaryotic genomes (release Dec-29-2020) using whole-genome based comparison in MiGA (Rodriguez-R et al., 2018).

### Pangenome Analysis

The NCBI-genome-download 0.2.12 script^3^ was used to retrieve all sequenced genomes from the genus *Sodalis* (March, 2020). The two strains obtained from deadwood were added to the set of genomes. *Sodalis*-like endosymbionts and other related strains analyzed previously by Santos-Garcia et al. (2017) were included to estimate size of the core gene set of *Sodalis*-allied group which takes into account significant genome reduction reported for some of these symbionts. Further analysis was focused on *Sodalis* strains with large genomes while those symbionts which are in the advanced reductive evolution process were excluded from the analysis because of their smaller genomes with significantly distinct gene compositions (Santos-Garcia et al., 2017). Selected genomes are summarized in Supplementary Table 1 and consist of *Sodalis* sensu stricto strains from unpublished as well as published studies (Toh et al., 2006; Chrudimský et al., 2012; Clayton et al., 2012; Oakeson et al., 2014; Rosas-Pérez et al., 2017; Rubin et al., 2018). Genomes were subsequently analyzed within pangenome analysis in anvi’o 6.2 (Eren et al., 2015, see the “Code Availability” section) to identify deadwood-associated genes and genes shared by all the genomes. ANI was calculated using pyANI 0.2.10 (Pritchard et al., 2015). The resulting image was processed with Inkscape^4^. For CAZyme annotation, prokka 1.13 gene prediction was used to obtain sequences of genes from 11 *Sodalis* genomes. The amino acid sequences were compared with the dbCAN database version 07312018 using run-dbcan.py 2.0.11 script (Zhang et al., 2018) and hmmer 3.3 (Eddy, 2011). CAZymes with an *e*-value ≤ 1E−20 were considered for further annotation. Ward’s clustering was used to group genomes based on the Hellinger transformed counts of all the detected CAZymes and pheatmap 1.0.12 package was used to generate a CAZy heatmap (Kolde, 2019). Spearman correlation was used to calculate genome-related statistics.

### *nifH* Phylogenetics

The curated database of *nifH* sequences classified to the phylum level or below was retrieved (Moynihan, 2020). Genes from the phylum *Proteobacteria* and a random *nifH* representative for each taxonomic group were selected. Two *nifH* genes from *Sodalis* sp. strain dw96 and three from *Sodalis ligni* were aligned with the retrieved *nifH* collection using the online MAFFT v7.475 (Katoh et al., 2018). Maximum likelihood tree was constructed using IQ-TREE v1.6.12 (-alrt 1000 -bb 1000) (Nguyen et al., 2014) using best-fit model GTR + F + R10 identified by ModelFinder (Kalyaanamoorthy et al., 2017) with UFBoot ultrafast bootstrap (Hoang et al., 2017). Tree was further edited using iTOL (Letunic and Bork, 2011).

### Habitat Context—Soil and Deadwood

To confirm the presence of *Sodalis* in deadwood 16S rRNA gene datasets from previous studies published by Hoppe et al. (2015), Moll et al. (2018), Probst et al. (2018), and Šamonil et al. (2020) were screened for similarity with the 16S rRNA genes of strain dw23^*T*^ and strain dw96 using BLAST 2.2.28+, and the relative abundance of the most abundant OTU within each study with >97% similarity to the cultivated strains was calculated.

Furthermore, three wood-associated *Sodalis* genomes together with genome of *Sodalis praecapticus* and genome of *Sodalis pierantonius* strain SOPE were used as a reference for the mapping of metagenomes of naturally decomposing wood obtained in the Žofínský prales Nature Reserve (Tláskal et al., 2021, unpublished). The metagenomic study included five age classes of differently decomposed deadwood of *Fagus sylvatica*, with the youngest age class decomposing for <5 years and the oldest age class decomposing for more than 41 years. *Sodalis ligni* dw23^*T*^ was recovered from the same decomposing tree as one of the metagenomic and metatranscriptomic sample (SRA BioSample SAMN13762420). Mapping procedure used BWA-MEM 0.7.17 (Li, 2013) with the default settings, samclip 0.4.0^5^ for the removal of mapped reads with hard- and soft-clipped flanking ends longer than 10 bp, samtools-1.9 (Li et al., 2009) for SAM/BAM format conversion and for filtering of mapped reads, and bedtools 2.29.2 (Quinlan and Hall, 2010) for the conversion of BAM into the BED format with the attached CIGAR string. Analysis was focused only on reads with mapping scores of 60, i.e., exact mapping to the unique position along the genome. The metagenomic sample SRR10968255 was omitted from mapping due to the low number of sequences.

The raw BWA-MEM 0.7.17 mapping files of the deadwood metagenomes to the *S. ligni* genome were used as inputs into the anvi’o 6.2 metagenomic pipeline to visualize the occurrence of *S. ligni* in individual deadwood age classes. No read filtering was applied to allow for single-nucleotide variants (SNVs) density calculation of *S. ligni* according to Reveillaud et al. (2019) (see “Code Availability” section). Similarly, metatranscriptomic reads from a previous study (Tláskal et al., 2021, unpublished) were mapped against the set of *S. ligni* genes to infer nitrogen fixation expression in decomposing wood. The data were summarized using R 4.0.0 (R Core Team, 2020) and tidyverse 1.3.0 package (Wickham et al., 2019). Significant differences were tested by the Kruskal-Wallis test using the agricolae package (de Mendiburu, 2017).

### Insect Cell-Free Cultivation and Enzyme Activity

To assess growth on insect cell-free media containing wood and to exclude the possibility that the *Sodalis* isolates are obligatory symbionts of a deadwood-associated insect, wood pellets from fresh beech wood were milled using an Ultra Centrifugal Mill ZM 200 (Retsch, Germany); 0.3 g of fine wood dust was mixed with 30 mL of Ringer solution and autoclaved. Media were inoculated in duplicate with *S. ligni* colonies growing on plates. Inoculated liquid media containing wood as the only C source were left at 24°C on an orbital shaker for incubation. Aliquots of media were regularly sampled, and samples were immediately frozen. To quantify the increase in cell biomass, DNA from the aliquots was extracted using a DNeasy UltraClean Microbial Kit (Qiagen, Germany) according to the manufacturer’s protocol with an additional initial spin for wood dust removal. The bacterial rRNA gene copies in DNA were quantified by qPCR using the 1108f and 1132r primers (Wilmotte et al., 1993; Amann et al., 1995). Sanger sequencing from the primer 1492r (Lane, 1991) was used to confirm the presence of *Sodalis*-only DNA in the final aliquots.

The activity of the cell wall-associated fraction of enzymes was measured in cell suspension after 2 weeks of incubation in 50 mL of liquid GY-VL55 medium as described previously (Lasa et al., 2019). For the catalase and oxidase tests, bacterial cells from a GY-VL55 agar plate were transferred into 3% H_2_O_2_ solution and 1% *N*, *N*, *N*’, *N*’-tetramethyl-*p*-phenylenediamine dihydrochloride drops, respectively. The Biolog PM1 system was used according to the manufacturer’s instructions to identify growth on different C sources.

## Results

*S. ligni* was able to grow on laboratory media in which beech wood was the only source of C, reaching a cell density up to 1.3 × 10^8^ mL^–1^ within 4 weeks of incubation. This growth excluded the possibility that it is an obligate symbiont of deadwood-associated insects. Hybrid assembly of the *Sodalis ligni* dw23^*T*^ provided 32 contigs (>200 bp, N50: 3.8 Mbp), a total genome size of 6.4 Mbp, GC content 54.96% and a mean coverage of 99×. Assembly of the *Sodalis* sp. strain dw96 provided 85 contigs (>200 bp, max 0.72 Mbp, N50: 0.25 Mbp), a total genome size of 5.9 Mbp, GC content 54.04% and a mean coverage of 52×. Genome completeness was 98.8 and 99.9% for the *Sodalis ligni* dw23^*T*^ and strain dw96, respectively. Contamination was not detected in the assembled genomes. *Sodalis ligni* dw23^*T*^ was placed within the genus *Sodalis* (*P* = 0.211) and belongs to a species not represented in the NCBI database of prokaryotic genomes (*P* = 0.0025).

**FIGURE 1:**
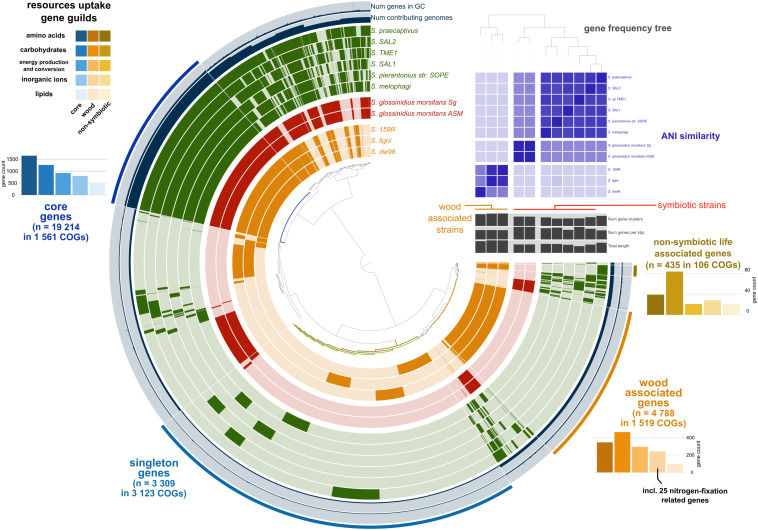
Pangenome analysis of the available *Sodalis* genomes. Genes (indicated by dark colors within the circles) were clustered based on orthologous similarity (inner tree), and genome layers were sorted based on a gene frequency tree (right upper tree). *Sodalis* guilds are color coded; deadwood-associated strains in orange, tsetse fly symbionts in red and insect symbionts with *S. praecaptivus* in green. ANI is indicated as the upper right heatmap. Pangenome analysis identified core genes shared by all genomes (bar plots in hues of blue), genes associated with non-symbiotic life (bar plots in hues of yellow) and genes associated with wood habitat (bar plots in hues of orange). Individual bar plots show counts of genes divided according to their function.

We retrieved 11 *Sodalis* genomes from NCBI together with the strain metadata (Supplementary Table 1). Pangenome analysis divided the available *Sodalis* genomes into three distinct guilds based on gene frequency (Figure 1). One group consisted of two genomes of *Sodalis glossinidius morsitans* (strains Sg and ASM), which are tsetse fly endosymbionts. The two *Sodalis* ligni dw23^*T*^ and strain dw96 isolated in this study from deadwood formed the second group together with the free-living *Sodalis* sp. strain 159R retrieved from NCBI as an anaerobic lignin degrader. The third group consisted of endosymbionts of hymenopteran, hemipteran, dipteran and coleopteran insects. The gene frequency, gene count and genome length of *S. praecaptivus* have high similarity with the third group of endosymbionts, thus placing this free-living strain distant from deadwood-associated *Sodalis* sp. strains dw23^*T*^ and dw96 despite their relatively high 16S rRNA gene similarity (97.2 and 97.8%, respectively). The ANI between strain dw23^*T*^ or strain dw96 and the rest of the analyzed genomes ranged from 76 to 78%, except for strain 159R (98 and 88%, respectively, Figure 1 and Supplementary Table 2). The ANI between strains dw23^*T*^ and dw96 was 88%. The genome size of all *Sodalis* strains and their gene count were closely correlated (ρ = 0.82, *P* = 0.004), while the genome size and gene counts per Kbp were negatively correlated (ρ = -0.63, *P* = 0.04). Free-living strains exhibited the lowest coding density (0.87–0.90 genes per kbp) and longer genes than symbionts (947 ± 7 bp and 732 ± 39 bp, respectively, *P* < 0.01, Supplementary Table 1).

**FIGURE 2:**
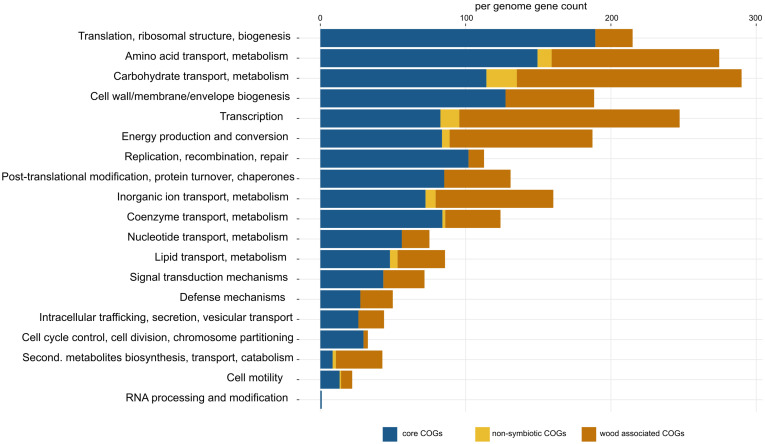
Detailed list of COG functions in *Sodalis* core genes (blue), non-symbiotic life-associated accessory genes (yellow) and wood-associated accessory genes (orange). Gene counts were corrected for the number of genomes in each group (core = 11, free-living = 4, wood-associated = 3). Per-genome numbers of additional genes with unknown function are not displayed, and their counts are: core = 403, free-living = 42, wood-associated = 651.

Pangenome analysis allowed us to identify clusters of orthologous genes (COGs) that were shared among strain guilds. Genes shared by all the genomes represent the core *Sodalis* pangenome (in average 1,747 genes per genome from 1,561 core gene clusters, 35.6% of genes from the average *Sodalis* genome, Figure 1). The core gene set shared with the *Sodalis*-like symbionts with extremely reduced genomes is represented by in average 130 genes from only 125 gene clusters. The COG categories with the highest share of classified core genes were translation, amino acid and carbohydrate metabolism, cell wall/membrane biosynthesis and replication categories (Figures 1, 2). Genes shared only among free-living strains were less frequent (in average 109 genes from 106 gene clusters; 2.0% per average free-living *Sodalis* genome) and were dominated by genes related to carbohydrate metabolism. Gene sharing among wood-associated strains was relatively common (in average 1,596 genes from 1,519 gene clusters, 28.4% per average wood-associated *Sodalis* genome), these genes were mostly classified in carbohydrate and amino acid utilization and transcription regulation COG categories. The nitrogenase gene *nifH* and genes encoding an ABC-type molybdate transport system were significantly enriched in the accessory genes of the wood-associated guild (adj. *q*-value = 0.01). *Sodalis ligni* dw23^*T*^, *Sodalis* sp. strain dw96 and *Sodalis* sp. strain 159R each contained *nifHDK* operons, while nitrogen fixation genes were absent in other *Sodalis* genomes. Similar to symbionts, wood-associated strains contained type III secretion system components.

**FIGURE 3:**
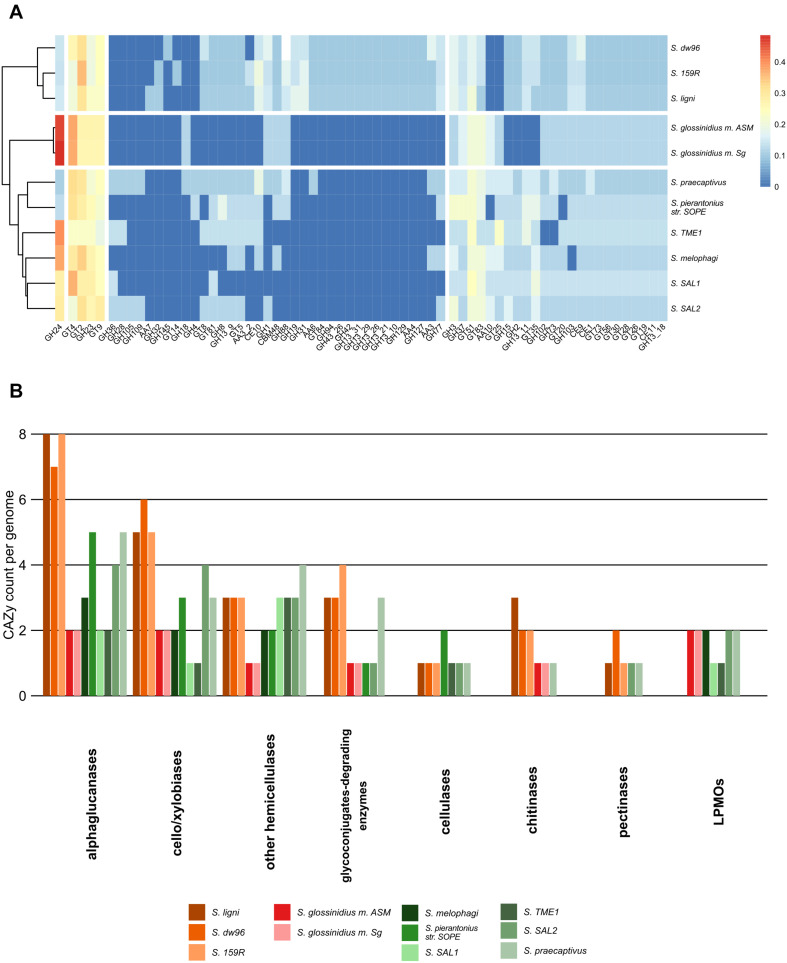
CAZy presence in the *Sodalis* genomes. **(A)** CAZy heatmap with clustering analysis shows the CAZyme abundance pattern, which follows *Sodalis* guild differentiation into wood-associated strains, tsetse symbionts and other symbiotic strains. Legend shows transformed counts. **(B)** Functional annotation of the CAZymes found in the genomes of *Sodalis* genomes. Genes for labile substrate utilization, such as cello/xylobiose and α-glucan, are increased in deadwood-associated strains.

In total, 833 genes encoding carbohydrate-active enzymes from 66 CAZy families were identified across all the genomes. Free-living strains together with the symbiotic *Sodalis* sp. strain SAL2 contained the highest per-genome share of CAZy, with >1.62% of the total genes being identified as CAZymes (Supplementary Tables 3, 4). Wood-associated strains showed the highest diversity of CAZy, possessing >80% of the detected CAZy families each. The lowest diversity of CAZy (43.9% of detected families) was recorded in tsetse fly-associated strains. CAZy richness in individual genomes was correlated with CAZy abundance (ρ = 0.7, *P* = 0.02). The distribution of detected CAZymes followed *Sodalis* guild differentiation, with distinct CAZy patterns for wood-associated strains, tsetse fly symbionts and other symbionts (Figure 3A). Additionally, the functional annotation of CAZy families showed to some extent their conservation within *Sodalis* guilds (Figure 3B and Supplementary Table 4). In comparison to symbionts, wood-associated strains had more genes for α-glucan, cellobiose, xylobiose and chitin utilization. Genes for cellulose, pectin and hemicelluloses degradation were also detected; however, the counts of these CAZymes were comparable with those in the genomes of symbionts. Glycoconjugate utilization CAZymes were enriched in wood dwellers as well as free-living *S. praecaptivus*. Lytic polysaccharide monooxygenases (LPMOs) were not detected in the genomes of wood-associated strains.

16S rRNA genes assigned to *Sodalis* were present in other published community studies from deadwood (see the “Data Availability” section) and typically exhibited high relative abundance. The mean abundance of one *Sodalis* OTU related to strains dw23^*T*^ and strain dw96 at 98.8% similarity was 2.79 ± 0.86% (*n* = 155, spruce deadwood in Finland, Schigel et al., unpublished data). Another *Sodalis* OTU (98% similarity to the presented genomes) was found on beech and spruce wood at 0.57–0.70% (Hoppe et al., 2015). When comparing deadwood of 13 tree species after 6 years of decomposition, the broadleaved species *Betula* sp., *Populus* spp., *Carpinus* sp. showed the highest abundance of *Sodalis* OTU (Supplementary Table 5; Moll et al., 2018). The abundance of other *Sodalis* OTU (99.6% similarity to the presented genomes) also differed in samples of spruce wood cubes (0.23 ± 0.16%, *n* = 26) and surrounding soil (0.004 ± 0.002%, *n* = 30, *P* < 0.001, Probst et al., 2018). Similarly, sequences assigned to *Sodalis* (100% similarity) were enriched in decomposing beech trees in Žofínský prales Nature Reserve (0.93 ± 0.48%, *n* = 24, *P* < 0.001), when compared to underlying soil (0.0004 ± 0.0002%, *n* = 48, Šamonil et al., 2020).

**FIGURE 4:**
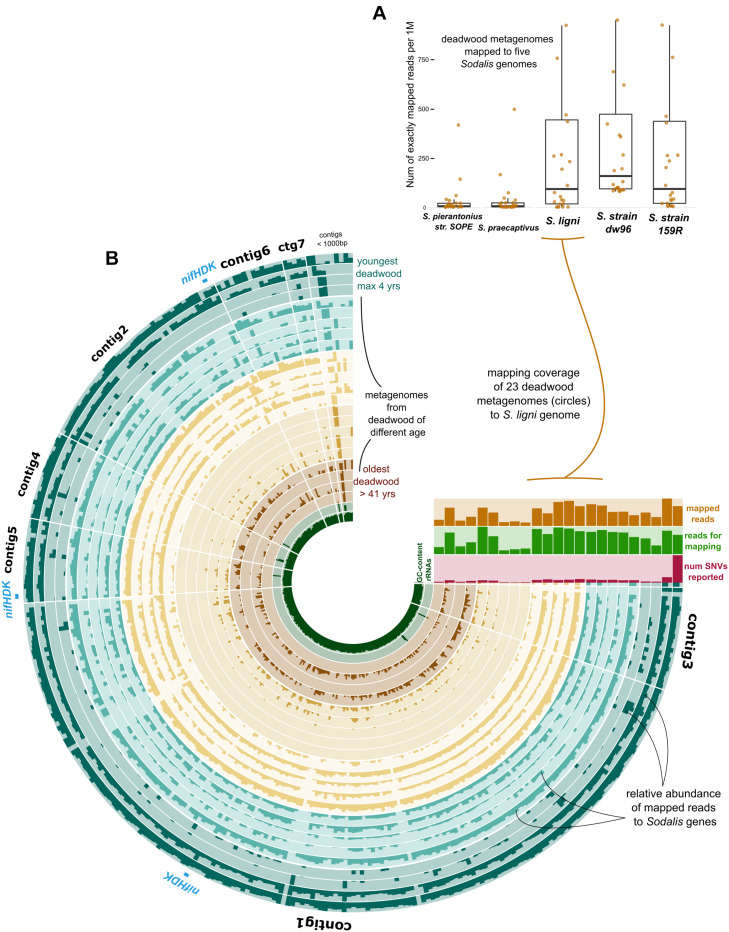
Deadwood metagenome mapping against the genome of *S. ligni*. **(A)** Boxplots show deadwood mapping count for wood-associated strains and *S. pierantonius* strain SOPE and *S. praecaptivus*. **(B)** The anvi’o plot with circles representing metagenome samples, the color coding of circles follows grouping of samples based on the length of deadwood decomposition. Dark bar plots within circles represent the relative abundance of reads mapped to *S. ligni* genes. Contigs were ordered based on the Euclidean distance and Ward linkage.

**FIGURE 5:**
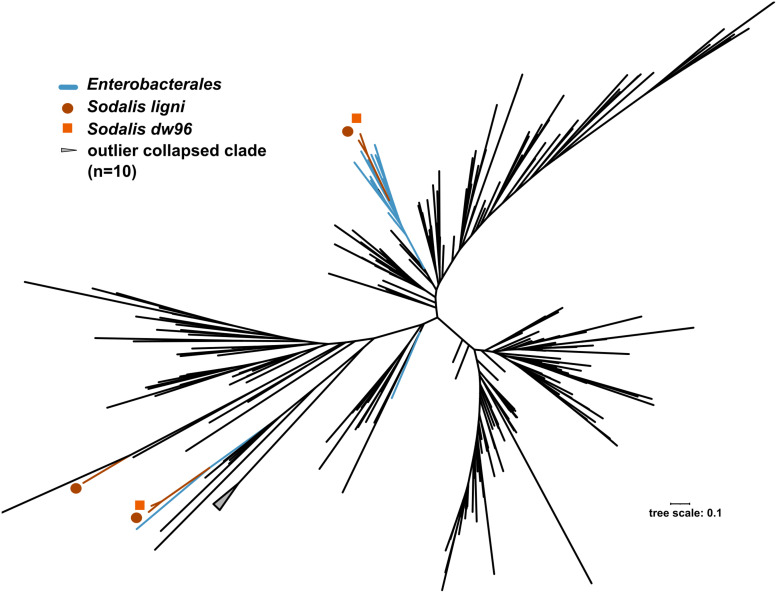
Phylogenetic tree based on *nifH* genes described from members of the phylum *Proteobacteria*. Blue branches denote *Enterobacterales*. Positions of the three *nifH* sequences from *Sodalis ligni* are labeled with dark brown circles, two of which are placed together with other enterobacteria. Two *nifH* sequences from *Sodalis* dw96 are labeled with bright brown squares, and both are placed together with other *nifH* from enterobacteria.

The exact mapping of the deadwood metagenomic data to the *Sodalis* genomes confirmed the higher abundances of the *S. ligni*, *Sodalis* sp. strain dw96 and *Sodalis* sp. strain 159R guild in a deadwood habitat than *S. praecaptivus* and *S. pierantonius* strain SOPE (*P* < 0.001, Figure 4A). For the three wood-associated strains, the average number of exactly mapped reads was 808 ± 220 reads per million reads (812 ± 397 reads per million for *S. ligni* only). *S. ligni* showed an even distribution throughout the gradient of deadwood decomposition stages when considering the proportion of mapped reads from the total reads available (Figure 4B). The rate of SNVs per one mapped read was 0.03 ± 0.01 (*n* = 23), and the density of SNVs against the *S. ligni* genome showed homogenous *Sodalis* populations in the deadwood metagenomes, with mean SNVs of 0.17 ± 0.07% except for the outlier population in one young deadwood metagenome (1.7% SNVs, Figure 4B). Most SNVs occurred in the third codon wobbling position (Supplementary Figure 1). Three distinct *nifHDK* operons were identified in the genome of *Sodalis ligni*. Two of these operons successfully called reads from two deadwood metatranscriptome samples (SRA accessions SRR10968245, SRR10968250). Notably, the same decomposing tree (BioSample SAMN13762420) served as the source of RNA for the latter metatranscriptome, as well as for the cultivation of *S. ligni*. Phylogenetic analysis of the *nifH* gene across the phylum *Proteobacteria* showed placement of *Sodalis* nitrogenase genes into distinct clades common to other *Enterobacterales*, with the exception of one *Sodalis ligni nifH* placed on a distant branch (Figure 5).

### Description of *Sodalis ligni* sp. nov.

*Sodalis ligni* (lig’ni. L. gen. n. *ligni* of wood, referring to the isolation of the type strain from wood material).

Cells are aerobic, Gram-negative, cocci (0.6 μm) or short rods (1.2 × 0.6 μm). They grow separately without forming groups. Colonies on solid medium are circular, regularly edged, smooth, mucoid and white. Catalase positive and oxidase negative. The enzymatic *in vitro* assays were positive for β-glucosidase, β-xylosidase, *N*-acetyl-glucosaminidase, phosphodiesterase, acid phosphatase and lipase. L-serine, D-saccharic acid, *N*-acetyl-beta-D-mannosamine, D-cellobiose, D-galactose, L-fucose, fumaric acid, L-lactic acid, β-methyl-D-glucoside, adonitol, dulcitol, glycerol, L-galactonic acid-γ-lactone, and monomethyl succinate could be used as C sources. Optimal growth occurs on GY-VL55 medium (Lladó et al., 2019) and GY agar at pH 4.5–5.5 at 24°C.

The type strain is dw23^*T*^ (=CECT 30299^*T*^ = BCCM/LMG 32208^*T*^), isolated from decomposing wood of *Fagus sylvatica* L. in a temperate mixed forest, Central Europe. The genomic G + C content of the type strain is 54.96%.

## Discussion

The genus *Sodalis* represents the model for the development of insect-associated lifestyle and host-symbiont interactions due to its evolutionarily young transition from a free-living to a symbiotic bacterium (Hall et al., 2020; Maire et al., 2020). Moreover, the tsetse fly symbiont *Sodalis glossinidius* is able to grow independently on its host under laboratory conditions, further pointing to the recent development of its non-facultative symbiosis (Goodhead et al., 2020). Until now, *S. praecaptivus* was the only free-living species described, which hints that there is a more diversified spectrum of life strategies than only association with insects (Clayton et al., 2012; Chari et al., 2015). Here, we describe two other free-living isolates from the *Sodalis* genus, one of which, strain dw23^*T*^, is suggested to represent the type strain for *Sodalis ligni* sp. nov. within the family *Enterobacterales*. The genome-to-genome alignment of the available *Sodalis* genomes shows ∼77% ANI with our strain, thus supporting the establishment of a new species within the *Sodalis* genus (Konstantinidis et al., 2006; Richter and Rosselló-Móra, 2009; Barco et al., 2020). This is supported also by comparison with NCBI database of prokaryotic genomes (Rodriguez-R et al., 2018). In contrast to *Sodalis* insect endosymbionts, *S. ligni* occupies deadwood and possesses genome characteristics connected with non-symbiotic life: a larger genome, a higher number of longer genes and a lower coding density (Toh et al., 2006; Oakeson et al., 2014). It refers to independent nutrient provisioning from more complex resources, the necessity for more sophisticated regulatory systems in fluctuating environments and a large effective population of free-living taxa in contrast to their symbiotic relatives (Giovannoni et al., 2014; Bobay and Ochman, 2018). We identified three ecological guilds within the genus *Sodalis*: tsetse endosymbionts, endosymbionts of other insect taxa and the wood-associated guild presented here. This differentiation is consistent based on ANI and on the functional genome content when considering either all genes or their subset CAZymes.

*S. ligni* appears in multiple 16S rRNA-based deadwood studies and is usually ranked among the most abundant taxa. Due to the multi-copy character of the 16S rRNA gene (Větrovský and Baldrian, 2013), amplicon studies might provide imperfect abundance estimates. This is true especially for the family *Enterobacterales*, as the mean number of 16S rRNA genes is 7 ± 0.5 (*n* = 3,130, Stoddard et al., 2015), which overestimates their abundance when comparing relative counts of 16S rRNA. Seven copies of 16S rRNA were also confirmed in the genome of *S. ligni*. Hybrid assembly using short and long reads helped to identify their individual copies, which are otherwise usually merged into one consensus copy by the assembler when assembling short reads only (Waters et al., 2018).

Despite the limitations of abundance estimates, amplicon studies showed clear habitat selection of *S. ligni*, which preferentially colonizes deadwood rather than the underlying soil (Probst et al., 2018; Šamonil et al., 2020). Moreover, *Sodalis*-related metagenome-assembled genomes are also absent in the recent large-scale cultivation-independent genome catalog, suggesting a low abundance of *S. ligni* in global soils (Nayfach et al., 2020). We therefore propose that *S. ligni* is a member of a wood-associated *Sodalis* guild. The independence of *S. ligni* from insect hosts is supported by its ability to grow solely on wood. The wood-associated guild further contains the other two isolates, *Sodalis* strain dw96 and *Sodalis* strain 159R, the former of which was identified in the present study and the latter was identified in an unpublished study. Other related *Sodalis* members, the previously described free-living *S. praecaptivus* and symbiont *Sodalis pierantonius* strain SOPE, do not show a deadwood preference (Oakeson et al., 2014). While for the symbiont this is not surprising, the main habitat and ecological functions of *S. praecaptivus* remain to be identified, considering its genome composition (which is closer to symbionts than to wood-associated strains), wood cultivation source and ability to grow in human and insect tissues (Clayton et al., 2012; Enomoto et al., 2017; Munoz et al., 2020). Based on the presence of virulence factors, *S. praecaptivus* may represent a plant pathogen transmitted by insects (Clayton et al., 2012) and thus may prefer living plants rather than decomposing wood.

Preferential colonization of wood of particular tree species by *S. ligni* might occur, as was observed in the data from conifers and broadleaved trees (Hoppe et al., 2015; Moll et al., 2018). However, further research across more deadwood types is needed to disentangle potential tree species selection. The abundance of *S. ligni* in decomposing wood does not seem to vary over tens of years, which is a typical lifetime for decomposing trees in temperate forests (Přívětivý et al., 2016), and the *S. ligni* population is homogenous with regard to SNVs density.

The core genes shared by all *Sodalis* strains contain genes for crucial cell functions, mainly translation, replication, transcription, amino acid and carbohydrate metabolism and cell wall/membrane biosynthesis. Furthermore, pangenome analysis unveiled a relatively low number of genes specific for non-symbiotic life, while accessory genes specific for wood-associated guild represent a significant portion of the total identified genes and are involved in, e.g., amino acid and carbohydrate metabolism. Such gene-specific enrichment is connected with a more diverse set of nutrient resources in deadwood habitats. The presence of type III secretion system genes in wood-associated strains might serve as an evolutionary predisposition for host infection by related symbiotic *Sodalis* members (Maire et al., 2020). The composition of CAZymes of wood-associated strains differentiates them from symbionts as well. Substrates targeted by CAZymes show that wood-associated *Sodalis* members utilize rather labile plant polymers or chitin to obtain C and do not serve as important cellulose degraders. The broader degradation capabilities were described for other deadwood-associated bacterial taxa such as those from the phyla *Acidobacteria* and *Bacteroidetes* (Tláskal and Baldrian, 2021). The ecological function of free-living *Sodalis* strains might be resolved based on the multiplied presence of *nifHDK* operons expressing nitrogenase, a key enzyme for nitrogen fixation which is dependent on the metal cofactors (e.g., molybdenum). The sequence difference between nitrogenase variants shows the importance of these genes for the bacterium. To reduce atmospheric nitrogen is energetically costly, and thus, there is probably a selection pressure in the *Sodalis* deadwood guild to not combine nitrogen fixation with resource-intensive degradation of recalcitrant plant polymers. Nitrogen fixation within the family *Enterobacterales* is known from taxa living in association with plants (Rosenblueth et al., 2004), with leaf-cutter ant fungus gardens (Pinto-Tomás et al., 2009) and with Diptera (Behar et al., 2005). Deadwood was previously described as a hotspot of nitrogen fixation by which bacteria enrich material with a low nitrogen content and relieve the nitrogen limitation on microbial colonization (Tláskal et al., 2021). Deadwood-associated *S. ligni* thus represents a candidate for nitrogen fixation and one of the rarely described free-living nitrogen fixing *Enterobacterales* members.

To conclude, we identified *S. ligni* as a member of the wood-associated *Sodalis* guild, and we provide a comprehensive analysis of its genome potential, substrate utilization and ecological function in the context of other known isolated *Sodalis* sensu stricto strains. Members of the *Sodalis* genus show broad life strategies with distinct specialization, indicating all-rounder character of this genus, including free-living deadwood inhabitants, non-symbiotic strains with the ability to colonize internal insect and human tissues and recently established symbionts in several insect orders. Such an extensive niche differentiation is known from another enterobacterial species, *Escherichia coli* (Tenaillon et al., 2010; Luo et al., 2011), but has not been described for *Sodalis*-related taxa. There is not enough evidence and more isolates from related sources are needed to determine whether deadwood served as a reservoir pool for symbiotic bacteria diversification via genome reduction or whether there was another common ancestor for both wood-associated and insect-associated guilds.

## Code Availability

The above methods indicate the programs used for analysis within the relevant sections. The code for reproducing all sequence processing is provided at https://github.com/TlaskalV/Deadwood-Sodalis.

## Data Availability Statement

Data described in this manuscript, including raw sequences from short-read sequencing and genome assembly files, have been deposited in the NCBI under BioProject accession numbers PRJNA599932 (*Sodalis ligni* dw23^T^) and PRJNA613885 (*Sodalis* sp. strain dw96). We further deposited MinION long-read sequencing data in ENA nucleotide archive (PRJEB43208), 16S rRNA sequences of *Sodalis* clustered in other deadwood studies (10.6084/m9.figshare.13227464), anvi’o pangenome databases (10.6084/m9.figshare.13221065), anvi’o deadwood metagenome mapping databases (10.6084/m9.figshare.13221074) and *nifH* tree in Newick format (10.6084/m9.figshare.14562279).

## Author Contributions

VT conceived the study with input from PB. VT performed the strain isolation, pangenome analysis, metagenomic mapping, and strain characterization. LŽ and VP performed the library preparation and sequencing, genome assembly, and annotation. VT and PB wrote the original version of the manuscript. All authors contributed to the final version of the manuscript.

## Conflict of Interest

The authors declare that the research was conducted in the absence of any commercial or financial relationships that could be construed as a potential conflict of interest.

## Footnotes

1 https://community.nanoporetech.com

2 https://github.com/rrwick/Porechop

3 https://github.com/kblin/ncbi-genome-download

4 https://inkscape.org/

5 https://github.com/tseemann/samclip

## Supplementary Material

Supplementary Table 1 List of available *Sodalis* genomes together with genome, life strategy metadata and description of referencing studies.

Supplementary Table 2 ANI statistics calculated for 11 available *Sodalis* genomes.

Supplementary Table 3 Total count, gene share and richness of CAZymes identified in *Sodalis* genomes. Deadwood-associated strains followed by *S. praecaptivus* contain the highest number of diverse CAZy.

Supplementary Table 4 Per family counts of CAZymes and their assigned activities in individual genomes.

Supplementary Table 5 Relative abundances of a *Sodalis* OTU with 99.6% similarity to *S. ligni* among deadwood of 13 tree species after 6 years of decomposition from the study Moll et al. (2018). Data represent means and standard errors.

Supplementary Figure 1 Single-nucleotide variants (SNVs) detected after mapping deadwood metagenome reads to the *S. ligni* genome shown as boxplots of the occurrence of SNVs for each codon position. Each dot represents one metagenome sample.
