# Supplementary figures and images for: Ecological Divergence Within the Enterobacterial Genus Sodalis: From Insect Symbionts to Inhabitants of Decomposing Deadwood
Source: Front Microbiol. 2021 Jun 11;12:668644. doi: 10.3389/fmicb.2021.668644 (PMC8226273; doi:10.3389/fmicb.2021.668644)

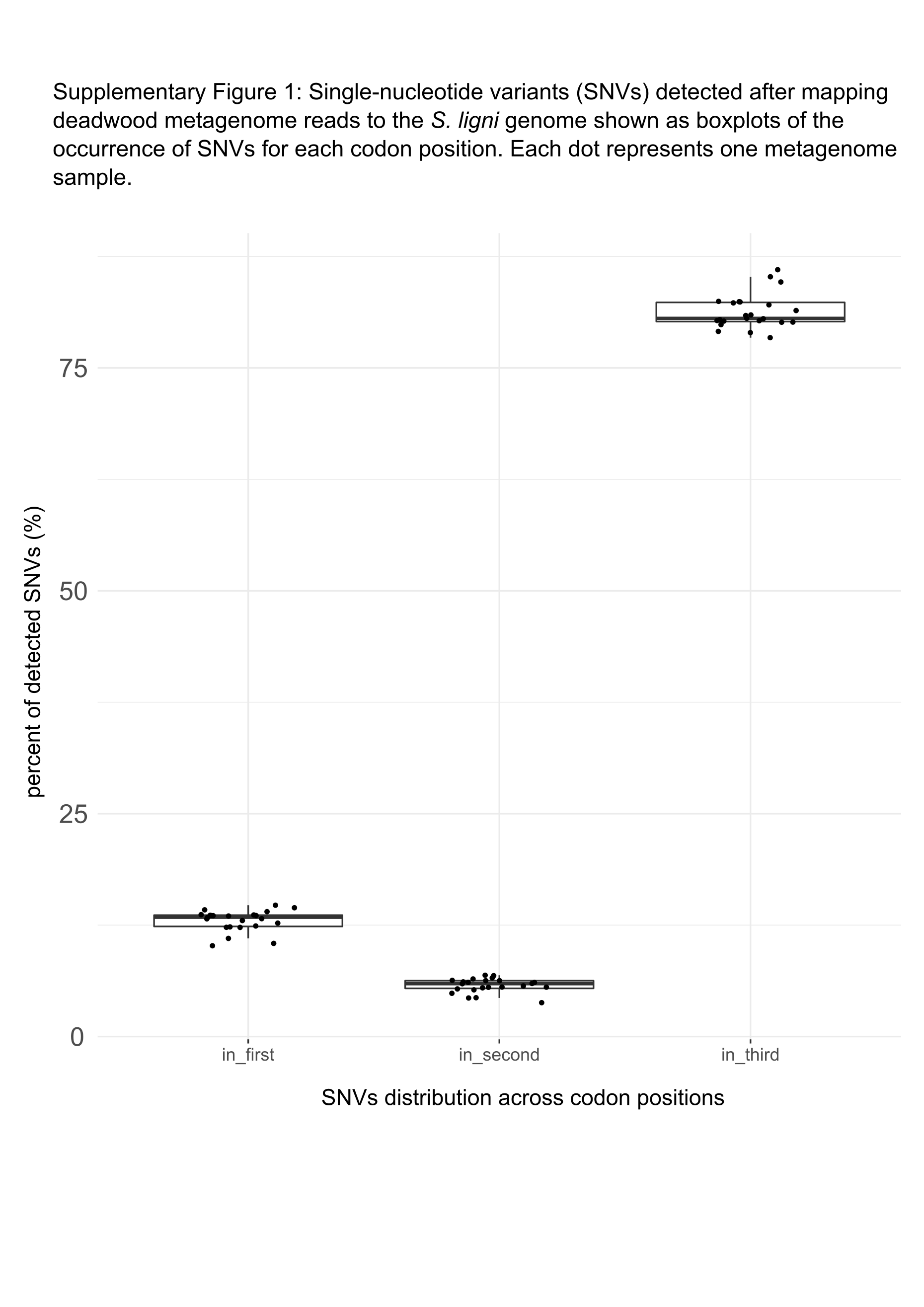

Supplement: Supplementary Figure 1 — Single-nucleotide variants (SNVs) detected after mapping deadwood metagenome reads to the S. ligni genome shown as boxplots of the occurrence of SNVs for each codon position. Each dot represents one metagenome sample. [file Image_1.TIFF]
